# Association of LSMx and LIMd Angles With Cephalometric Dental and Soft Tissue Parameters of Incisor Proclination and Lip Protrusion

**DOI:** 10.7759/cureus.67761

**Published:** 2024-08-25

**Authors:** Erum Amin, Anum Tariq, Ramsha Nawaz, Alaina Tariq, Bushra Gul

**Affiliations:** 1 Orthodontics, Armed Forces Institute of Dentistry, Rawalpindi, PAK

**Keywords:** mentolabial, cephalometry, nasolabial, incisor position, lip position

## Abstract

Background: Soft tissue analysis can be used to assess anatomical features but may or may not accurately correlate with underlying hard tissue morphology, leading to an incorrect perception of malocclusion.

Objective: This study aimed to assess the reliability of different soft tissue reference lines used to evaluate anteroposterior lip position and the position of incisors and malocclusion and compare it with those assessed via hard tissue angles (LSMx and LIMd) and determine if they are true indicators of underlying protrusion of incisors and malocclusion.

Method: A total of 120 pre-treatment lateral cephalometric radiographs were selected where patients were 18-30 years old, diagnosed as Skeletal Class I, II (Division 1 and 2), and III malocclusion. The measurements taken were SN to point A angle (SNA), SN to point B angle (SNB), angle between point A and point B (ANB), upper incisor to SN plane angle (UI-SN), upper incisor to palatal plane angle (UI-PP), incisor mandibular plane angle (IMPA), Ricketts' E line, Sushner's S2 line, nasolabial (NL) angle, mentolabial (ML) angle, LSMx angle, and LIMd angle.

Results: In the Class I malocclusion group, when the upper lip was assessed, the distribution of UI-SN, UI-PP, E line to UL, S line to UL, NL angle, and LMax was significantly different statistically (p=0.000), though the assessment of lower variables in Class I malocclusion showed the distribution of IMPA, E line to UL, S line to UL, ML angle, and LMand angle has a statistically significant difference (p=0.007). In Class II Division 2 malocclusion, a significant difference was observed for the upper variables (p=0.000), whereas the distribution of lower values was the same across all the variables (p=0.0724). In the sample of Class III malocclusion, a significant correlation was found in the upper variables, while the distribution among lower variables did not show any significant difference (p=0.211).

Conclusion: This study indicates that the upper and lower soft tissue correlation with hard tissue variables is reliable for some variables but not throughout for all. Soft tissue analysis (under study) can be used to assess disproportion, but it fails to correlate to the underlying hard tissue morphology and does not explain the correct malocclusion. Further studies based on 3D diagnosis to formulate a close relationship are encouraged that can help assess soft and hard tissue patterns consistent with one another.

## Introduction

Lip prominence is an important determinant of facial esthetics, which in turn reflects underlying dental and skeletal patterns [1]. Lip projection, lip symmetry, and lip height are crucial factors for attractive labial appearance [2]. Many patients who are not satisfied with their labial appearance seek orthodontic treatment. Therefore, the assessment of lip prominence and competency is an essential part of orthodontic examination and treatment planning.

Lip projection varies for different ethnicities. Sushner [3] conducted a soft tissue study on African Americans and concluded that African Americans have more protrusive lips compared to Caucasians, whereas white ethnicities have thinner lips. Now in the 21st century, esthetic trends have shifted towards fuller lips as depicted by fashion magazines and all forms of media, especially for females [4]. These trends in facial esthetics are sadly derived from the judgment of the public and driven by public opinion. They then trickle down and make their way into the domain of arts, movie stars, fashion models, and beauty pageant contests and influence the masses. To improve facial esthetics, an orthodontist must be aware of what the layman considers an ideal profile [5].

There are different methods for measuring lip prominence, which include Ricketts' E line, the S1 line of Steiner, the B line of Burstone, the Holdaway H line, and Sushner's S2 line. Other ways to assess lip position include true vertical, nasolabial (NL), and mentolabial (ML) angles, and even more accurate ways would be through projections to the pterygomaxillary vertical line as proposed by Nanda et al. [6]. These measurements help form a treatment plan for an orthodontic patient and aid in the decision of extraction or non-extraction treatment plan.

Relative projection of lips in the horizontal plane is affected by lip thickness, while there is only limited literature on the role of tooth position affecting lip projection. A study by Feghali et al. [7] concluded that there is a relaxed postural lip position which is independent or partially independent of tooth position, whereas another study conducted by Mehta et al. [8] on the Indonesian population showed that there were significant changes in lip position after the retraction of anterior teeth in patients with bimaxillary proclinations. Due to conflicting evidence on the role of tooth position on lip projection, further studies are required that can correlate this relationship. This also highlights the importance of assessing comparisons between various existing parameters that assess the soft tissue profile.

The role of lips as an etiological factor in causing various malocclusions is also well-cited in the literature. Class II Division 1 patients are known to have lower lip traps causing an increased overjet. Class II Division 2 patients typically have strap-like lower lips and high resting lower lip lines due to short anterior facial height. Class III malocclusion patients are characterized by full and pendulous lower lip. Thus, malocclusion can also affect the relative projection of lips [9].

The objectives of the study were to assess the reliability of different soft tissue reference lines (E line, S line, S2 line) to evaluate anterior-posterior lip position and inclination of incisors and malocclusion, to compare anterior-posterior lip positions assessed via different soft tissue reference lines with those assessed via soft tissue angles (NL angle, ML angle), to compare anterior-posterior lip positions assessed via different soft tissue reference lines with those assessed via hard tissue angles (LSMx and LIMd), and to assess and compare if soft tissue reference lines, soft tissue angles, and hard tissue angles (LSMx and LIMd) are true indicators of underlying protrusion of incisors and malocclusion.

## Materials and methods

This comparative cross-sectional study was conducted in the orthodontics department of the Armed Forces Institute of Dentistry (AFID), Rawalpindi, Pakistan, after approval from the institution's ethical committee (approval number: 918/Trg dated May 13, 2020). Data included digital lateral cephalograms of patients, who were seeking orthodontic treatment. Patients fulfilling our inclusion criteria were selected on a non-probability consecutive pattern. The sample size was calculated by using the WHO calculator, with a population prevalence proportion of 90%, yielding a sample size of 138 patients. The formula used was n=z2p (1-p)/d2, where z=1.96, d=5.5%, and p=90%. Out of the calculated sample size, 18 cephalograms were dropped out because of selection bias and confounding variables. Finally, a total of 120 pre-treatment lateral cephalometric radiographs were selected that fulfilled the inclusion criteria.

The selected head films were taken from the same X-ray machine (CS 8000C Carestream, France) in the same institution; it was made sure by two observers that the selected films were of good quality and taken in natural head position (NHP) with lips that were in the rest position.

All tracings were done on acetate paper. A pilot test was performed on 20 randomly selected radiographs from the data within 12 parameters, to check intra-observer reliability. No differences in tracings were found. An intra-observer calibration test was performed, where the same radiographs were traced two weeks later and then an inter-observer calibration test was performed by the co-author of this study. There were no differences in terms of the cephalometric tracing of landmarks, and a calculated reading was found.

Inclusion criteria were patients with an age range of 18-30 years, diagnosed as Skeletal Class I, II (Division 1 and 2), and III malocclusion. Subjects with a history of orthodontic or orthognathic treatment, any existing craniofacial anomaly, strained lips, and chin, and in whom at least one skeletal measurement did not match, were excluded.

**Figure 1:**
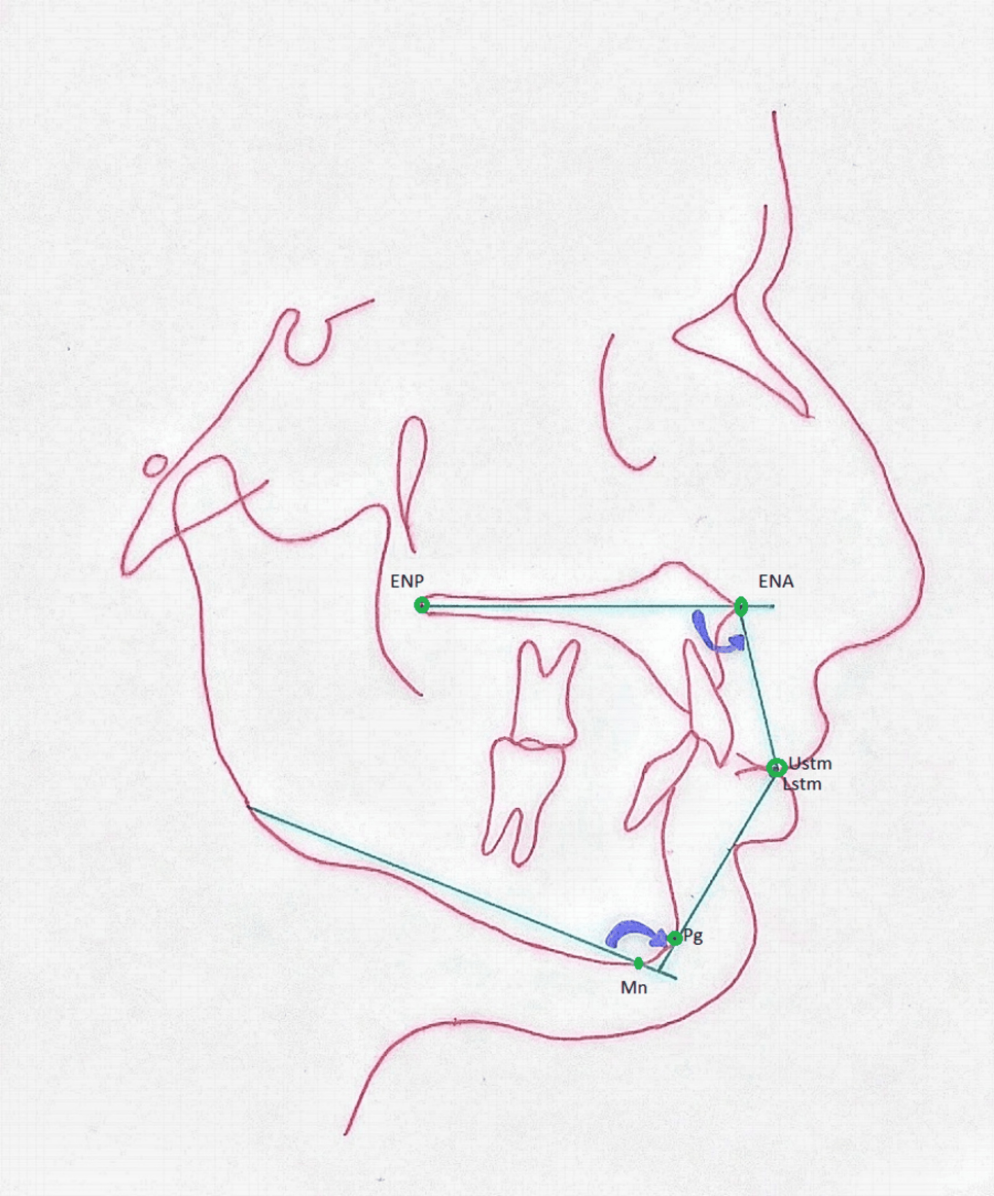
Proposed angles to assess the anteroposterior position of the upper and lower lip Ustm: stomion of the upper lip; Lstm: pogonion to estomion of the lower lip; Pg: pogonion; Mn: menton; Go: gonion Reference: [6]

Data was analyzed statistically using IBM SPSS Statistics for Windows, Version 26.0 (Released 2019; IBM Corp., Armonk, New York, United States). The Shapiro-Wilk test was performed, and data was not found to be normally distributed. Effect modifiers such as gender and age were controlled via stratification. Pearson's chi-squared test was used to understand the relationship between variables across the four groups (Class I, Class II Division 1, Class II Division 2, and Class III) and gender. Non-parametric Friedman's two-way analysis of variance by ranks was selected to assess differences and make pairwise comparisons among these groups after adequately ranking the values as normal, protrusive, or retrusive (based on the already established norms). P<0.05 was taken as significant. For better assessment and more accurate results, projections to pterygomaxillary vertical lines proposed by Nanda et al. (as shown in Figure 1) were used [6].

Frequencies and percentages were calculated for quantitative analysis. The normal value ranges of the parameters recorded in the study are shown in Table 1.

**Table 1:** Normal ranges of cephalometric parameters included in the study SNA: SN to point A angle; SNB: SN to point B angle; ANB: angle between point A and point B; UI-SN: upper incisor to SN plane angle; UI-PP: upper incisor to palatal plane angle; IMPA: incisor mandibular plane angle

Parameter	Values
SNA	80±2°
SNB	78±2°
ANB	0-4°
UI-SN	102±4°
UI-PP	108±4°
IMPA	90±4°
Ricketts' E line	Upper lip: -3±2; lower lip: -2±2
Nasolabial angle	90-110°
Mentolabial angle	90-100°
LSMx angle	105.5±5.5°
LIMd angle	88±5.5°
Sushner's S2 line (female)	Upper lip: 8.8 mm; lower lip: 6.7 mm
Sushner's S2 line (male)	Upper lip: 10.3 mm; lower lip: 7.8 mm

## Results

Out of 120 patients, most of them were female (65.8%). The mean age of the total sample was 21.87±3.49, out of which the mean age of male patients was 21.27±3.24, while that of female patients was 22.18±3.59. Statistically, no significant difference was observed based on gender.

Table 2 shows the categorization of all four malocclusion groups indicating that all the variables were statistically significant. In the Class I malocclusion group, when the upper lip was assessed, the distribution of UI-SN, UI-PP, E line to UL, S line to UL, NL angle, and LMax was significantly different statistically (p=0.000). The pairwise comparison further revealed significant differences between S line to UL and E line to UL (p=0.045), S line to UL and UI-SN plane (p=0.004), S line to UL and UI-PP (p=0.003), NL angle and both UISN (p=0.013) and UIPP (p=0.011), LMax and UI-SN (p=0.019), as well as LMax to UI-PP (p=0.016) as shown in Table 2 and Table 3.

**Table 2:** Rank-wise comparison of different variables among four malocclusion groups (upper arch) n: number of cases; N: normal; P: protrusive; R: retrusive; A: acute; O: obtuse Pearson's chi-squared (cross-tabulation) test; UI-SN: upper incisor to SN plane angle; UI-PP: upper incisor to palatal plane angle; NL: nasolabial; UL: upper lip

Malocclusion	UI-SN (n=120)			UI-PP (n=120)			E line-UL (n=120)			S line-UL (n=120)			NL angle (n=120)			LMax-UL (n=120)		
Soft tissue profile	N %	P %	R %	N %	P %	R %	N %	P %	R %	N %	P %	R %	N %	A %	O %	N %	P %	R %
Class I	16.7	73.3	10.0	16.7	73.3	10.0	36.7	43.3	20.0	63.3	36.7	0.0	3.3	86.7	10.0	60.0	23.3	16.7
Class II Division 1	10.0	90.0	0.0	13.3	86.7	0.0	63.3	33.3	3,3	30.0	70.0	0.0	6.7	83.3	10.0	26.7	36.7	36.7
Class II Division 2	10.0	0.0	90.0	13.3	3.3	83.3	40.0	13.3	46.7	46.7	26.7	26.7	13.3	70.0	16.7	43.3	6.7	50.0
Class III	23.3	70.0	6.7	16.7	76.7	6.7	26.7	10.0	63.3	46.7	26.7	26.7	16.7	33.3	50.0	53.3	30.0	16.7
Total	15	58.3	26.7	15.0	60.0	25.0	41.7	25.0	33.3	46.7	40.0	13.3	10.0	68.3	21.7	45.8	24.2	30.0
Pearson's chi-squared test	0.000*			0.000*			0.000*			0.000*			0.070*			0.006*		

**Table 3:** Rank-wise comparison of different variables among four malocclusion groups (lower arch) n: number of cases; N: normal; P: protrusive; R: retrusive; A: acute; O: obtuse Pearson's chi-squared (cross-tabulation) test; IMPA: incisor mandibular plane angle; ML: mentolabial; LL: lower lip

Malocclusion	IMPA (n=120)			E line-LL (n=120)			S line-LL (n=120)			ML angle (n=120)			LMand (n=120)		
Class I	33.3	60.0	6.7	33.3	46.7	20.0	40.0	53.3	6.7	60.0	33.3	6.7	53.3	33.3	13.3
Class II Division 1	6.7	80.0	13.3	26.7	56.7	16.7	43.3	53.3	3.3	70.0	13.3	16.7	50.0	43.3	6.7
Class II Division 2	20.0	50.0	30.0	43.3	6.7	50.0	43.3	23.3	33.3	53.3	33.3	13.3	20.0	76.7	3.3
Class III	16.7	46.7	36.7	33.3	26.7	40.0	26.7	53.3	20.0	53.3	46.7	0.0	20.0	50.0	30.0
Total	19.2	59.2	21.7	34.2	34.2	31.7	38.3	45.8	15.8	59.2	31.7	9.2	35.8	50.8	13.3
Pearson's chi-squared test	0.008*			0.002*			0.011			0.000*			0.001*		

On the assessment of lower lip variables in Class I malocclusion, the distribution of IMPA, E line-UL, S line-UL, ML angle, and LMand angle showed statistically significant differences (p=0.007). Pairwise comparison showed that a significant difference was observed between LMand and ML angle (p=0.009) and between S line-LL and ML angle (p=0.025).

The distribution of UI-SN, UI-PP, E line, S line, and NL angle and LMax variables in Class II Division 1 malocclusion was not the same across groups statistically (p=0.000). A significant difference was found between E line-UL and UI-PP (p=0.005), E line-UL and UI-SN (p=0.003), E line-UL and LMax (p=0.001), NL angle and UI-PP (p=0.010), NL angle and UI-SN (p=0.006), and NL angle and LMax (p=0.002) on the pairwise comparison. The statistical analysis of lower values revealed significant differences in LMand and ML angle (p=0.013), LMand and IMPA (p=0.007), S line and ML angle (p=0.025), and S line-LL and IMPA (p=0.014).

In Class II Division 2 malocclusion, a significant difference was observed for upper variables (p=0.000), and the pairwise comparison of the sample revealed differences between NL and UI-PP (p=0.000), NL-UISN (p=0.000), S line-UL and UI-PP (p=0.001), S line-UL and UI-SN (p=0.001), and S line-UL and UI-SN (p=0.000). The values for E line-UL and UI-PP, E line-UL and UI-SN, and LMax and UI-PP were p=0.016, p=0.006, and p=0.017, respectively. The distribution of lower values was the same across all the variables; thus, the null hypothesis was accepted (p=0.0724). 

In the sample of Class III malocclusion, a significant correlation was found between NL angle and UI-PP (p=0.027), NL and E line-UL (p=0.000), LMax and E line-UP (p=0.005), and S line and E line-UL (p=0.049). However, the distribution among lower variables did not show any significant difference (p=0.211) as shown in Table 4 and Table 5.

**Table 4:** Pairwise comparison of variables among different malocclusion groups (upper arch) Sig.: significance; *: p<0.05, Friedman two-way analysis of variance by rank; NL: nasolabial; UI-PP: upper incisor to palatal plane angle; UI-SN: upper incisor to SN plane angle; UL: upper lip

Variables	Class I		Class II Division 1		Class II Division 2		Class III	
Sample 1 to sample 2	Test statistics	Sig.	Test statistics	Sig.	Test statistics	Sig.	Test statistics	Sig.
NL to S line-UL	-0.183	0.704	0.817	0.091	0.233	0.629	0.783	0.105
NL to E line-UL	0.783	0.105	-0.100	0.836	0.717	0.138	1.733	0.000*
NL to LMax	-0.067	0.890	-1.500	0.002*	-0.733	0.129	-0.383	0.427
NL to UI-PP	1.233	0.011*	1.250	0.010*	1.883	0.000*	1.067	.0027*
NL to UI-SN	1.200	0.013*	1.333	0.006*	2.033	0.000*	0.933	0.053
S line-UL to E line-UL	0.967	0.045*	-0.917	0.058	0.483	0.317	0.950	0.049*
S line-UL to LMax	-0.250	0.605	-0.683	0.157	-0.500	0.301	0.400	0.408
S line-UL to UI-PP	1.417	0.003*	0.433	0.370	1.650	0.001*	0.283	0.558
S line-UL to UI-SN	1.383	0.004*	0.517	0.285	1.800	0.000*	0.150	0.756
E line-UL to LMax	0.717	0.138	-1.600	0.001*	-0.017	0.972	1.350	0.005*
E line-UP to UI-PP	0.450	0.352	1.350	0.005*	1.167	0.016*	-0.667	0.168
E line-UP to UI-SN	0.417	0.388	1.433	0.003*	1.317	0.006*	-0.800	0.098
LMax to UI-PP	1.167	0.016*	-0.250	0.605	1.150	0.017*	0.683	0.157
LMax to UI-SN	1.133	0.019*	-0.167	0.730	1.300	0.007*	0.550	0.255
UI-PP to UI-SN	-0.033	0.945	0.083	0.863	0.150	0.756	-0.133	0.783

**Table 5:** Pairwise comparison of variables among different malocclusion groups (lower arch) Sig.: significance; *: p<0.05, Friedman two-way analysis of variance by rank; IMPA: incisor mandibular plane angle; ML: mentolabial; LL: lower lip

Variables	Class I		Class II Division 1		Class II Division 2		Class III	
Sample 1 to sample 2	Test statistics	Sig.	Test statistics	Sig.	Test statistics	Sig.	Test statistics	Sig.
LMand to S line-LL	0.150	0.713	0.100	0.806	Insignificant (0.724)		Insignificant (0.211)	
LMand to IMPA	0.317	0.438	1.100	0.007*				
LMand to E line-LL	0.550	0.178	0.617	0.131				
LMand to ML	1.067	0.009*	1.017	0.013*				
S line-LL to IMPA	0.167	0.683	1.000	0.014*				
S line-LL to E line-LL	0.400	0.327	0.517	0.206				
S line-LL to ML	-0.917	0.025*	-0.917	0.025*				
IMPA to E line-LL	-0.233	0.568	0.483	0.236				
IMPA to ML	-0.750	0.066	0.083	0.838				
E line-LL to ML	-0.517	0.206	-0.400	0.327				

## Discussion

It is a well-known fact that variations exist among individuals, be it hard tissue or soft tissue. The effect of underlying skeletal morphology on soft tissue configuration demands special attention when it comes to assessment for treatment planning purposes. As numerous methods are available to carry out the analysis of lip position, lip thickness, and tooth inclination, the current study focused on including various (soft and hard tissue) parameters to obtain a clearer vision of the variables that may accurately depict soft and hard tissue morphology for all existing malocclusion groups as soft tissue harmony holds great importance during planning and executing a successful orthodontic treatment. Soft tissue discrepancies can have a significant impact on hard tissue measurements, as there is a close relationship between the two in cephalometric analysis.

Sushner's line which passes through the stable nasion has been identified to show maximum consistency, making it the de facto line for sagittal lip positions when doing profile analysis. Statistical analysis of the S2 line shows a statistically insignificant difference between males and females for upper and lower lip protrusion. Umale et al. [10], in their study, arrived at a similar conclusion with no significant difference in relation to the S2 line between males and females. On the contrary, a comparable study [11] has been conducted where five different reference lines were used to assess lip position. Males showed prominence in both the upper and lower lip compared to females for the S2 line. Values for our study are more in line with those of the Caucasian population where there was no statistically significant difference in lower lip protrusion between both genders. This study was conducted for North Indians, where a differentiating factor between our population and theirs could be posteriorly positioned forehead and nasion in Indians [12].

As parameters under study are formed or calculated using different landmarks, great variability exists in the values; therefore, first categories were made based on the standardized ranges, and then pairwise comparisons were made and analyzed. One of the most common issues during manual cephalometric tracing is human error, which can be corrected using digital analysis. These landmarks, however, still must be located manually. With the arrival of artificial intelligence in the realm of dentistry, automated location and cephalometric analysis have shown high interrater reliability [13].

Analysis of maxillary parameters exhibited great variation between soft and hard tissue structures. Likewise, in a study conducted by Singh et al. [14], there was an insignificant correlation between upper incisors and upper lip. A study by Oswal et al. [15] also confirmed the same; when assessing 30 patients with Class II Division 1 malocclusion, they found that the soft tissue profile was not an accurate representation of the underlying skeletal profile during orthodontic treatment. On the contrary, Namratha et al. [16] concluded a significant relationship between the upper lip and maxillary incisors in a sample of 80 patients in a predominantly South Indian population.

The NL angle is a highly significant measurement that is formed by the juncture of the anterior upper lip and columella at the subnasale. Its mean value can be taken as 102±8°. Supported underneath by hard tissues, it is surprising that the value for this angle alone does not represent sufficient information about which specific parameter has resulted in its variability [17]. Likewise, in the present study, the NL angle failed to correspond to the proclination or retroclination of the patient's respective dental component. However, in another study by Devine et al. [18], the effect of incisor retraction was observed in Class II Division 1 patients treated with and without extractions on the NL angle with respect to the nasal and labial components individually. The study revealed that upper lip retraction was responsible for an overwhelming 90% of the NL angle increase, whereas a mere 10% was related to the change of inclination of the lower border of the nose.

Similarly, E line, S line, and LMax were inconsistent in correlating hard and soft tissue discrepancy, with the soft tissue profile being an unreliable representative of underlying hard tissue anatomy. This can be seen in a similar study by Singh et al. [14], which evaluated changes in facial profile relative to upper and lower incisors. They found that facial parameters from U1 to L1 were found to be incoordinate in relation to hard tissue status in 86.8% of their sample and normal in only 13.2%. These findings are consistent with those observed by Kochel et al. [19], who also found that various changes can be made in the growth phase in the hard tissue parameters of the patient.

Furthermore, similar findings were reported by Shayani et al. [20], who also found that facial imbalance occurred due to both maxillary and mandibular hard tissue parameters at different patient chronological and skeletal ages.

Lower values were much more consistent in their findings as ML and E line depicted the same results consistent with lower inclinations. The same results were seen in the study conducted by Murthy et al. [21], with a difference between the E line only in between classes (I, II, III). The LMand and S line were inconsistent for Class I and Class II Division 1. These findings contradict the study by Garg et al. [22], who determined the response of the lower lip to maxillary and mandibular incisor position. They found that in the static state, the vertical dimension of lower facial height and lower incisor position did not relate to changes in the lower lip. Limited sample size and absence of placebo are limitations of the study.

## Conclusions

This study indicates that the upper and lower soft tissue correlation with hard tissue variables is reliable for some variables but not throughout for all. Soft tissue analysis (under study) can be used to assess disproportion, but it fails to correlate to the underlying hard tissue morphology and does not explain the correct malocclusion. Since the paradigm has shifted to soft tissue, therefore, latest imaging techniques that give a 3D approach to studying the pattern of malocclusion should be made part of treatment planning. Further studies are encouraged based on 3D diagnosis to formulate a close relationship that can help assess soft and hard tissue patterns consistent with one another.
